# Clinical characteristics and outcome of SARS-CoV-2 infection in admitted patients with chronic lymphocytic leukemia from a single European country

**DOI:** 10.1186/s40164-020-00195-x

**Published:** 2020-12-18

**Authors:** Ana Muntañola, Guillermo Villacampa, José Ángel Hernández-Rivas, Rosalía Alonso, Fátima Mirás, Santiago Osorio, Mónica Baile, Patricia Baltasar, Javier López Jiménez, Ines Hernandez-Rodriguez, Susana Valenciano, Ana Alfayate, Eva Gimeno, Abelardo Bárez, Ana C. Oliveira, Rosalía Riaza, Pilar Romero, Julio Delgado, Lucrecia Yáñez, Amaya Zabalza, Ana Torres, Mª Isabel Gómez-Roncero, Marta Crespo, Raúl Córdoba, Juan José Mateos-Mazón, Sonia Pérez, Rafael Andreu, Jorge Labrador, Mª Elena Ruiz, César Andrés Velasquez, Mª José Terol, Raquel Santiago, Mª Jesús Vidal, Fiz Campoy García, Lucía Villalón, Begoña S. Muiña, Joan Alfons Soler, Cristina Seri, Mª José Sánchez, Amalia Cuesta, Rafael Ramos, Adrián Sánchez-Montalvá, Isabel Ruiz-Camps, Marcos González, Pau Abrisqueta, Francesc Bosch, of the GELLC (Grupo Español de Leucemia Linfática Crónica)

**Affiliations:** 1 Hospital Universitari Mútua Terrassa, Barcelona, Spain; 2 Oncology Data Science, Vall D’Hebron Institute of Oncology, Barcelona, Spain; 3 SOLTI Breast Cancer Research Group, Barcelona, Spain; 4 Hospital Universitario Infanta Leonor, Madrid, Spain; 5 Hospital Universitario Puerta de Hierro, Madrid, Spain; 6 Hospital Universitario 12 de Octubre, Madrid, Spain; 7 Hospital Universitario Gregorio Marañón/Gregorio Marañón Health Institute (IiSGM), Madrid, Spain; 8 Hospital Universitario de Salamanca/IBSAL, CIBERONC and Center for Cancer Research-IBMCC (USAL-CSIC), Salamanca, Spain; 9 Hospital Universitario La Paz, Madrid, Spain; 10 Hospital Universitario Ramón y Cajal, Madrid, Spain; 11 Hospital Germans Trias i Pujol-ICO Badalona, Barcelona, Spain; 12 Hospital Universitario Príncipe de Asturias, Madrid, Spain; 13 Hospital Clínico San Carlos, Madrid, Spain; 14 Hospital del Mar, Barcelona, Spain; 15 Complejo Asistencial de Ávila, Ávila, Spain; 16 Hospital Duran i Reynals-ICO Hospitalet, Barcelona, Spain; 17 Hospital Universitario Severo Ochoa, Madrid, Spain; 18 Hospital Universitario Donostia, San Sebastián, Spain; 19 Hospital Clínic de Barcelona, Barcelona, Spain; 20 Hospital Universitario Marqués de Valdecilla, Santander, Spain; 21 Complejo Hospitalario de Navarra, Pamplona, Spain; 22 Complejo Asistencial de Segovia, Segovia, Spain; 23 Hospital Virgen de la Salud, Toledo, Spain; 24 Department of Hematology, Vall d’Hebron Barcelona Hospital Campus, Passeig de la Vall d’Hebron, 119-129, 08035 Barcelona, Spain; 25 Hospital Universitario Fundación Jiménez Díaz, Madrid, Spain; 26 Hospital Universitario de Cruces, Barakaldo, Spain; 27 Hospital Clínico Universitario de Valladolid, Valladolid, Spain; 28 Hospital Universitario La Fe de Valencia, Valencia, Spain; 29 Hospital Universitario de Burgos, Burgos, Spain; 30 Hospital Universitario del Tajo, Madrid, Spain; 31 Hospital de Mollet, Barcelona, Spain; 32 Hospital Clínico Universitario de Valencia, Valencia, Spain; 33 Hospital Sant Joan de Déu de Manresa - Fundació ALTHAIA, Barcelona, Spain; 34 Hospital Universitario de León, León, Spain; 35 Complexo Hospitalario Universitario de Ourense, Ourense, Spain; 36 Hospital Universitario Fundación Alcorcón, Madrid, Spain; 37 Hospital Rafael Méndez de Lorca, Murcia, Spain; 38 Consorci Corporació Sanitària Parc Taulí de Sabadell, Barcelona, Spain; 39 Hospital Central de la Defensa Gómez Ulla, Madrid, Spain; 40 Hospital Universitario Lucus Augusti, Lugo, Spain; 41 Hospital Sierrallana (Torrelavega), Cantabria, Spain; 42 Hospital Universitario de Badajoz, Badajoz, Spain

## Letter to the editor

Spain has been one the most affected countries by the severe acute respiratory syndrome coronavirus 2 (SARS-CoV-2), the causative agent of coronavirus disease 2019 (COVID-19) pandemic [1, 2]. Patients with chronic lymphocytic leukemia (CLL) could be at risk of more severe COVID-19 clinical forms [3] since they often carry immune perturbations aggravated by treatments used for the disease itself [4]. Two major series on patients with COVID-19 and CLL encompassing different countries and health systems reported heterogeneous factors related to the outcome [5, 6]. Herein, we are presenting the largest series of CLL patients with proved COVID-19 from a single country and Health system.

We identified 165 patients with CLL and COVID-19 across 40 Spanish centers (Additional file 1: Table S1 and S2) between March 1, 2020 and May 31, 2020. In summary, at the time of infection median age was 73 years, 27% were younger than 65, and 40% had comorbidities (CIRS ≥ 6). Eighty-five patients (52%) were in watch & wait (W&W), 34 (21%) had been previously treated, whereas 46 patients (28%) were currently on CLL treatment, mainly with BTK inhibitors (BTKi) (n = 34) and venetoclax (n = 7). Increased CRP (> 0.3 mg/dL) was detected in 27.8%, increased D-dimer (> 500 mg/mL) in 70%, ferritin > 400 mg/mL in 74%, and elevated IL-6 in 88% of patients in whom it was assessed. Among these inflammatory parameters, ferritin and D-dimer were significantly lower in patients receiving BTKi at the time of COVID-19 compared to the others (Additional file 1: Fig. S1). 92% of the patients required hospital admission, with 31% requiring intensive management.

**Fig. 1:**
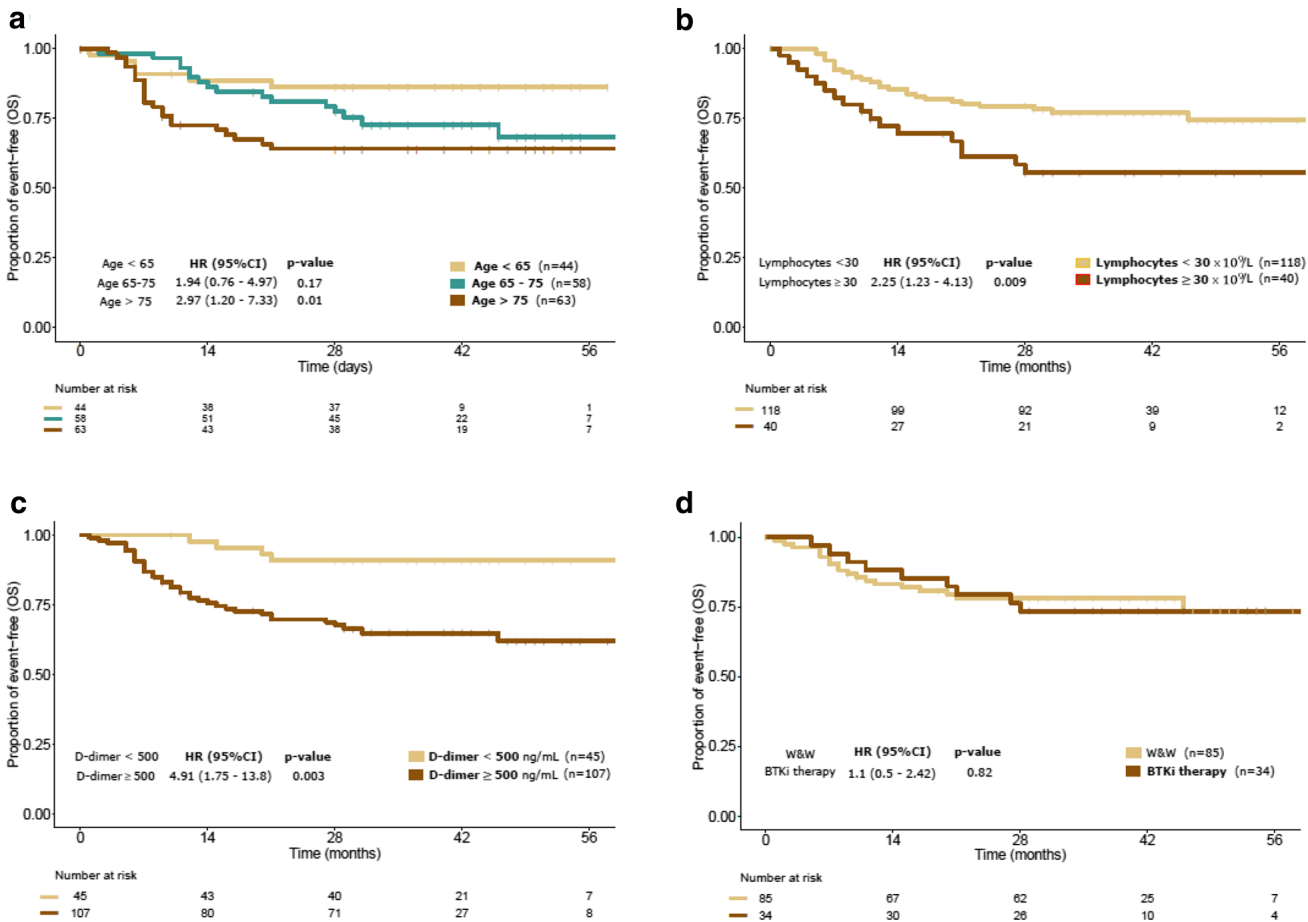
Overall survival curves according to (**a**) age, (**b**) Lymphocyte count, (**c**) D-dimer levels, and (**d**) BTKi treatment

Regarding survival, 45 deaths (27%) were observed, all of them due to SARS-CoV-2 infection. The case fatality rate (CFR) for admitted patients was 33.6% and the OS estimates of the entire cohort at 28 days was 74.1%. In the univariate analysis, age, CIRS ≥ 6, Binet stage B-C, hemoglobin < 10 g/dL, lymphocytosis (≥ 30 × 10^9^/L), CRP, and D-dimer levels were associated with OS. At the multivariate analysis, age (HR = 1.36, [95% CI 1–1.86]), lymphocytosis (HR = 1.96, [95% CI 1.05–3.63]), and D-dimer (HR = 4.35, [95% CI 1.53–12.3]) maintained its independent statistical significance (Fig. 1 & Additional file 1: Table S3). Patients on W&W presented similar OS than patients receiving an active CLL-directed therapy. Notably, treatment with BTKi (n = 34) did not influence mortality of the infection in comparison with patients on W&W (HR: 1.1 [CI 95% 0.5–2.42]; Fig. 1d).

**Fig. 2:**
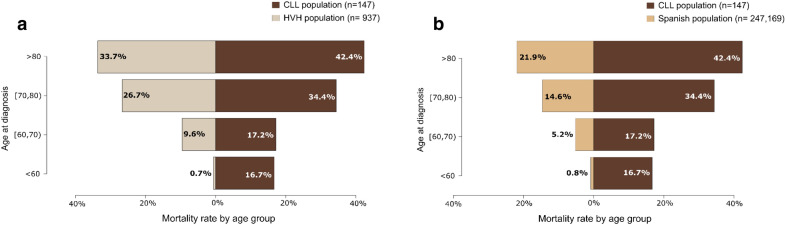
Mortality rates in different groups of age comparing admitted patients with CLL to (**a**) patients admitted at University Hospital Vall d’Hebron Campus, Barcelona and (**b**) general population diagnosed with COVID19 in Spain

Mortality rate by segments of age was contrasted with 937 patients admitted for COVID-19 at University Hospital Vall d’Hebron, excluding patients with hematologic malignancies, solid tumors, or with other causes of immunosuppression. Overall, mortality rates were higher in CLL patients (adjusted OR = 1.74 [95% CI 1.14–2.65], p = 0.01). The major difference between both cohorts was observed in the higher mortality for CLL patients < 60 years (16.7% vs 0.7%, p < 0.001). Of note, three out of the four young CLL patients that died were untreated and lack comorbidities, suggesting a negative effect of CLL in those patients. Finally, mortality was significantly higher compared to the mortality rates by age reported by the Spanish Ministry of Health up to May 2020 (adjusted OR = 3.91 [95% CI 2.70–5.66], p < 0.001) (Fig. 2 and Additional file 1: Table S4).

The inferior survival observed in patients with CLL and COVID-19 in our series seems to be similar to the data reported in other series of patients with hematologic malignancies, pointing to a patient population more vulnerable to COVID-19 infection [7].

In summary, in our series mortality rate was 27%, with a CFR for admitted patients of 33.6%, resembling the ones reported by Mato et al. [5] and by the ERIC/CLL Campus series [6]. In contrast to the observations reported by the ERIC/CLL Campus series, and in agreement with Mato et al. [5], age and comorbidities were strongly related to mortality. Importantly, and as opposed to the two former mentioned series [5, 6], lymphocytosis was associated with OS, suggesting that a more active CLL disease at the time of infection could increase the vulnerability to COVID-19. The inflammatory parameters analyzed in our series corelated with COVID-19 outcome. Thus, increased CRP and D-dimer predicted for a shorter survival, the latter maintaining its adverse prognostic impact in the multivariate analysis. Finally, in our series and in agreement with Mato et al. [5] patients receiving BTKi at the time of COVID-19 presented similar outcome as patients never treated and, accordingly exhibit lower levels of ferritin and D-dimer, which could be in part explained by the suggested protective role of BTKi against SARS-CoV-2 infection severity due to its immune-modulator effect [8–10].

## Supplementary Information

**Additional file 1: Table S1.** Patient and CLL features at the time of COVID-19 infection (n = 165). **Table S2.** COVID-19 manifestations, management, and outcomes. **Table S3.** Univariate and multivariate OS analysis of baseline characteristics. **Table S4.** Characteristics of patients infected by SARS-CoV-2 i) treated at University Hospital Vall d’Hebron and ii) overall Spanish population. **Figure S1.** Levels of inflammatory parameters according to treatment with BTKi. (* indicates p < 0.05).
