# Supplementary material for: Clinical characteristics and outcome of SARS-CoV-2 infection in admitted patients with chronic lymphocytic leukemia from a single European country
Source: Exp Hematol Oncol. 2020 Dec 18;9:37. doi: 10.1186/s40164-020-00195-x (PMC7746919; doi:10.1186/s40164-020-00195-x)

**SUPPLEMENTAL MATERIAL**

**PATIENTS & METHODS**

We retrospectively collected data from 165 CLL patients consecutively diagnosed with SARS-CoV-2 infection since March 2020 to May 2020, when pandemic outbreak emerged in Spain, in 40 centers belonging to the GELLC group (Grupo Español de Leucemia Linfática Crónica). Only patients with a previous diagnosis of CLL and proved SARS-CoV-2 infection by positive PCR or serological test were included in the study. For all cases, clinical data from CLL diagnosis and treatment were collected through an electronic form. Shortly, these data included sex, age, RAI/Binet stage, *IGHV* mutational status and details about previous therapies for CLL. Regarding COVID-19 infection diagnosis, we collected clinical symptoms, blood examinations, date of PCR and serologic tests, radiology findings, treatments for the infection and clinical outcome.

Thresholds used for inflammatory parameters analyzed in the series (ferritin, D-dimer and IL-6) were set according to the upper limit value of normality commonly used in local laboratories from the centers of the study. For CRP, we based our threshold in previous data of CPR and COVID-19.^1^ Patients were classified as never treated (watch & wait), previously treated but not receiving CLL-directed therapy at the time of COVID-19, and patients with active treatment during the infection. SARS-CoV-2 infection was considered severe when the patient required non-invasive mechanical ventilation or tracheal intubation, or when the patient died due to COVID-19. Outcomes of COVID-19 were classified as resolved, death or remain admitted. Case fatality rate (CFR) was calculated considering resolved and dead cases.

Data collected from CLL patients was compared with data obtained from admitted patients at the University Hospital Vall d’Hebron during the SARS-CoV-2 outbreak (submitted) from March 1, 2020 and May 31, 2020. Information for patients infected by SARS-CoV-2 was available in 1185903 patients, but 248 patients were finally excluded from the analysis for the following reasons: diagnosis of hematologic malignancies (n=61), solid tumors (n=127), patients with other causes of immunosuppression such as organ transplant recipients and HIV patients (n=52), and non-complete information (n=8). In addition, data on CLL patients was also compared with the official data on severity and mortality regularly reported by the Spanish Ministry of Public Health.^2^ (Table S4)

The study was approved by the PETHEMA Foundation, by the Ethics Committee at University Hospital Vall d’Hebron, and by the Agencia Española del Medicamento (AEMPS) where it was considered observational. The Ethics Committee stated that patient’s informed consent was not required, as the study was considered a priority issue concerning public health.

**Statistical analysis**

A descriptive analysis of all baseline characteristics at CLL diagnosis and at SARS-CoV-2 infection was performed. Continuous variables were expressed as median and ranges, and categorical variables were expressed as absolute values and percentages. Overall survival (OS) was defined as time from SARS-CoV-2 infection to death or last follow-up. Survival analysis was calculated using the Kaplan–Meier method and the log-rank test for statistical comparison. Cox proportional hazard models were used to obtain hazard ratios (HRs) with 95% CIs. For variable selection in multivariate analysis, we used the least absolute shrinkage and selection operator (LASSO)~~lasso~~ method to construct the most parsimonious model. The mortality rate due to COVID-19 in CLL population was compared to general population using the Cochran–Mantel–Haenszel test after adjusting for age groups to calculate adjusted odds ratios (OR). Categorical variables were studied using the Fisher exact test. Two-sided p values < 0.05 were considered as statistically significant. All analyses were undertaken using R statistical software version 3.6.2.

**Bibliography**

1. Tan C, Huang Y, Shi F, et al. C‐reactive protein correlates with computed tomographic findings and predicts severe COVID‐19 early. Journal of Medical Virology. 2020.

2. Gobierno de España. Ministerio de Sanidad. https://cnecovid.isciii.es/covid19/.

**SUPPLEMENTAL TABLES & FIGURES**

**Table S1.** Patient and CLL features at the time of COVID-19 infection (n=165)

| General Characteristics |  | n (%) |
| --- | --- | --- |

| Age, median (range) | 73 (37-94) |
| --- | --- |
| Follow-up time in days, median (range) | 29 (1-74) |

| Gender | 59 (36% female) |
| --- | --- |
| Unmutated IGHV (n=77) | 45 (58) |
| Comorbidities  CIRS > 6  Hypertension  Diabetes Mellitus  Chronic Obstructive Pulmonary Disease  Current smoker  Former smoker | 66 (40)  76 (46)  36 (22)  14 (8)  12 (7)  42 (25) |
| Clinical Stage*  RAI (n=113)  0-I  II  III-IV  BINET (n=114)  A  B  C | 90 (80)  5 (4)  18 (16)  90 (79)  9 (8)  15 (13) |
| CLL treatment history n (%) | |
| Never treated (W&W) | 85 (52) |
| Prior therapy   - Lines of therapy for previously treated, median (range) | 80 (48)  1 (1-7) |
| - Last CLL treatment finished prior COVID-19^¥^   Ibrutinib  FCR  Bendamustine - Rituximab  Chlorambucil - Rituximab  Chlorambucil - Obinutuzumab  Chlorambucil  Rituximab  Not reported  Months from end of treatment to COVID-19, median (range) | 34 (21)  4 (12)  12 (35)  6 (17)  2 (6)  4 (12)  1 (3)  3 (9)  2 (6)  29 (0.3-196.1) |
| - Ongoing CLL-directed therapy during COVID-19   Ibrutinib  Venetoclax  Acalabrutinib  Zanubrutinib  Alkylating agents  Chlorambucil - Obinutuzumab  Corticosteroids | 46 (28)  30 (65)  7 (16)  3 (7)  1 (2)  2 (4)  2 (4)  1 (2) |

CIRS: cumulative illness rating scale; FCR: fludarabine, cyclophosphamide and rituximab; W&W: watch & wait; *Only for W&W and patients not receiving CLL-directed therapy at the time of COVID; **^¥^**Patients previously treated not receiving CLL-directed therapy at the time of COVID-19.

**Table S2**. COVID-19 manifestations, management, and outcomes.

| **Patient’s characteristics at SARS-CoV-2 infection n (%)** | | | | | | |
| --- | --- | --- | --- | --- | --- | --- |
| CLL treatment during COVID-19 course  Held  Continued  Ibrutinib  Venetoclax  Acalabrutinib  Corticosteroids | | 37 (80)  9 (20)  6  1  1  1 | | | | |
| **Clinical Symptoms n (%) n (%)** | | | | | | |
| Fever | 144 (87) | Diarrhea | | | 44 (27) | |
| Cough | 117 (71) | Rhinorrhea | | | 10 (6) | |
| Anosmia | 11 (7) | Dyspnea | | | 101 (61) | |
| Dysgeusia | 9 (5) | Sickness /vomiting | | | 24 (14) | |
| Headache | 24 (15) | Myalgia | | | 62 (38) | |
| Encephalitis | 1 (0.6) | Thrombosis | | | 10 (6) | |
| Skin lesions | 6 (4) | Myocarditis | | | 2 (1.3) | |
| **Hematology Blood Tests** | | | | | | |
| Hemoglobin > 10 (g/dL) | 137 /159 (86) | Lymphocytes (x10^9^/L)  < 1 | | | n=159  29 (18) | |
| Platelets > 100 x 10^9^/L | 135/159 (84) | 1-30 | | | 90 (57) | |
| WBC > 25 x 10^9^/L | 54/158 (34) | ≥ 30 | 40 (25) | | | |
| **Biochemistry Tests n (%)** | | | | | | |
| CRP > 0.3 mg/dL | 152/158 (96) | Ferritin > 400 ng/mL | | 85/115 (74) | | |
| D-dimer > 500 ng/mL | 107/152 (70) | IL-6 > 7 pg/mL | | 59/67 (88) | | |
| Procalcitonin > 2ug/L | 6/91 (7) | Gamma Globulins < 7 g/L | | 27/50 (54) | | |
| **SARS-CoV-2 therapy n (%) n (%)** | | | | | | |
| Antibiotherapy | 146 (88) | Hydroxychloroquine | | | | 151 (92) |
| Cephalosporins 18 (12)  Macrolides 26 (18)  Amoxicilin 9 (6)  Antibiotic combination 86 (59)  Intravenous Gamma Globulins 10/85 (12)  LMWH 113 (70) | | Lopinavir/Ritonavir | | | | 101 (61) |
|  |  | Remdesivir | | | | 4 (2) |
|  |  | Tocilizumab | | | | 49 (30) |
|  |  | Anakinra | | | | 14 (8) |
|  |  | Corticosteroids | | | | 102 (62) |
| **COVID-19 management** | **n (%)** | | | | | |
| Outpatient  Admitted   - No supplemental oxygen - Supplemental oxygen - Non-invasive mechanical ventilation - Orotracheal intubation / ECMO | | 13 (8)  152 (92)  12 (8)  92 (61)  26 (17)  22 (14) | | | | |
| Secondary infections | | 31 (19) | | | | |
| **Survival Outcome** | |  | | | | |
| Resolved  Remain admitted  Death | | 102 (62)  18 (11)  45 (27) | | | | |

*Patients not currently on CLL treatment at the time of COVID-19; CRP: C Reactive Protein; LMWH: low-molecular-weight heparin; ECMO: Extracorporeal membrane oxygenation

**Table S3.** Univariate and multivariate OS analysis of baseline characteristics.

|  | Overall survival analysis (n=165, events=45) | | | | |
| --- | --- | --- | --- | --- | --- |
|  |  | Univariate analysis | | Multivariate analysis | |
|  | % mortality | HR 95% CI | P value | HR 95% CI | P value |
| **Age** (10 years increment) | 27.3% | 1.53 (1.15 – 2.04) | **0.004** | 1.36 (1 – 1.86) | **0.05** |
| **Sex** |  |  |  |  |  |
| Female  Male | 25.4%  28.3% | Ref.  1.21 (0.60 – 2.08) | -  0.72 | -  - | -  - |
| **CIRS** |  |  |  |  |  |
| < 6  ≥ 6 | 19.2%  39.4% | Ref.  2.24 (1.24 – 4.05) | -  **0.008** | Ref.  1.64 (0.89 – 3.02) | -  0.11 |
| **Smoker** |  |  |  |  |  |
| No  Current or ex-smoker | 25.2%  31.5% | Ref.  1.3 (0.71 – 2.38) | -  0.39 | -  - | -  - |
| **Hypertension** |  |  |  |  |  |
| No  Yes | 25.8%  28.9% | Ref.  1.19 (0.66 – 2.13) | -  0.56 | -  - | -  - |
| **COPC** |  |  |  |  |  |
| No  Yes | 26.5%  35.7% | Ref.  1.45 (0.57 – 3.68) | -  0.56 | -  - | -  - |
| **IGHV** |  |  |  |  |  |
| Mutated  Unmutated | 28.9%  28.1% | Ref.  1.04 (0.45 – 2.44) | -  0.92 | -  - | -  - |
| **Binet (n=119)***  A  B - C | 23.3%  45.8% | Ref.  2.18 (1.05 – 4.53) | -  **0.03** | -  - | -  - |
| **Numbers of line**  0  1  2+ | 23.3%  30.3%  32.6% | Ref.  1.28 (0.60 – 2.73)  1.33 (0.68 – 2.61) | -  0.53  0.40 | -  - | -  - |
| **IL-6** |  |  |  |  |  |
| < 7 pg/mL | 25% | Ref. | - | - | - |
| ≥ 7 pg/mL | 20.3% | 0.81 (0.18 – 3.61) | 0.78 | - | - |
| **CLL therapy**  W&W  Ongoing BTKi therapy | 22.4%  26.5% | Ref.  1.1 (0.5 – 2.42) | -  0.82 | -  - | -  - |
| **Hb level**  < 10 g/dL  ≥ 10 g/dL | 45.8%  25% | Ref.  0.48 (0.24 – 0.94) | -  **0.03** | -  - | -  - |
| **Platelet count**  < 100x10^9^/L  ≥ 100x10^9^/L | 33.3%  27.3% | Ref.  0.79 (0.38 – 1.64) | -  0.53 | -  - | -  - |
| **Lymphocyte count**  < 30 x10^9^/L  ≥ 30 x10^9^/L | 22.9%  42.5% | Ref.  2.25 (1.23 – 4.13) | -  **0.009** | Ref.  1.96 (1.05 – 3.63) | -  **0.03** |
| **Lymphopenia**  < 1 x10^9^/L | 39.3% | Ref. | - | - | - |
| ≥ 1 x10^9^/L | 26% | 0.64 (0.32 – 1.26) | 0.2 | - | - |
| **CRP**  < 0.3 mg/dL  ≥ 0.3 mg/dL | 24%  43.2% | Ref.  1.99 (1.09 – 3.61) | -  **0.02** | Ref.  1.64 (0.89 – 3.02) | -  0.11 |
| **D-dimer**  < 500 ng/mL  ≥ 500 ng/mL | 8.9%  35.5% | Ref.  4.91 (1.75 – 13.8) | -  **0.003** | Ref.  4.35 (1.53 – 12.3) | -  **0.006** |
| **Ferritin** |  |  |  |  |  |
| < 400 ng/mL  ≥ 400 ng/mL | 33.3%  25.9% | Ref.  0.79 (0.38 – 1.68) | -  0.55 | -  - | -  - |

*Patients not currently on CLL treatment at the time of COVID-19; CRP: C Reactive Protein

**Table S4.** Characteristics of patients infected by SARS-CoV-2 i) treated at University Hospital Vall d’Hebron and ii) overall Spanish population

|  | | **University Hospital Vall d’Hebron** | **Overall Spanish population** |
| --- | --- | --- | --- |
| **Total, n** | | 937 | 247,169 |
| **Age at diagnosis, n (%)** | | 405 (43.2%)  178 (19%)  176 (18.8%)  178 (19%) | 120,019 (48.6%)  35,074 (14.2%)  33,345 (13.5%)  58,731 (23.6%) |
|  | < 60 y |  |  |
|  | [60,70) y  [70,80) y |  |  |
|  | $\geq$ 80 y |  |  |
| **Sex (female) , n (%)** | | 408/934 (43.7%) | 140,606 (56.9%) |
| **Current or former smoker** | | 214/925 (23.1%) | - |
| **ECOG** $\boldsymbol{\geq}$ **1** | | 145/900 (16.1%) | - |
| **Diabetes** | | 174/925 (18.8%) | - |
| **D-dimer**$\boldsymbol{\geq}$ **500 ng/mL** | | 159/678 (23.5%) | - |
| **Hemoglobin** $\boldsymbol{\geq}$ **10 (g/dL)** | | 870/902 (96.5%) | - |
| **Admitted patients, n (%)** | | 937 (100%) | 91,878 (37.2%) |
| **Admitted at ICU, n (%)** | | 170 (18.1%) | 7,675 (3.1%) |

**Supplemental Figure S1**

**Figure S1**. Levels of inflammatory parameters according to treatment with BTKi.

(* indicates p<0.05)


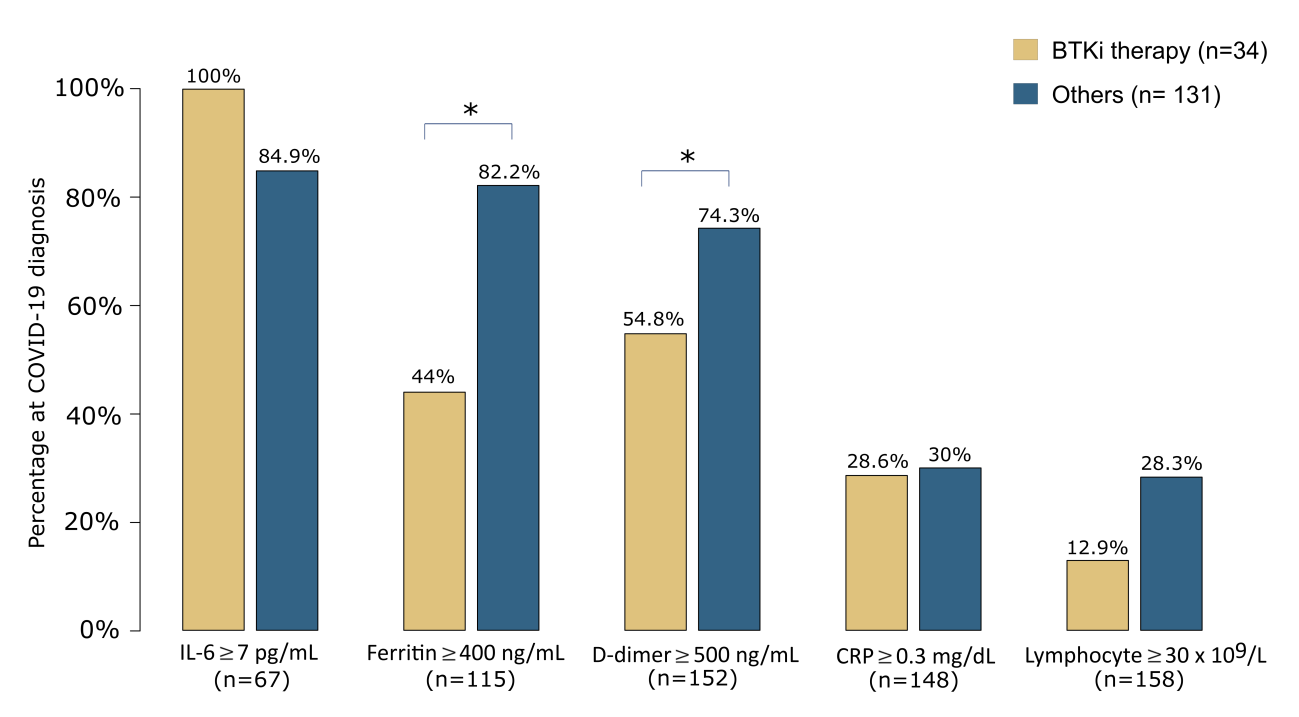

Supplement: Supplementary file 1 — Additional file 1: Table S1. Patient and CLL features at the time of COVID-19 infection (n = 165). Table S2. COVID-19 manifestations, management, and outcomes. Table S3. Univariate and multivariate OS analysis of baseline characteristics. Table S4. Characteristics of patients infected by SARS-CoV-2 i) treated at University Hospital Vall d’Hebron and ii) overall Spanish population. Figure S1. Levels of inflammatory parameters according to treatment with BTKi. (* indicates p < 0.05). [file 40164_2020_195_MOESM1_ESM.docx]
